# Identifying Outpatient-Specialized High-Volume Prescribers of Antibiotics in Older Adult Populations – Philadelphia, PA 2021

**DOI:** 10.1017/ash.2024.96

**Published:** 2024-09-16

**Authors:** Giovanny Zapata, Jenna Scully, Tiina Peritz, Jane Gould

**Affiliations:** Philadelphia Department of Public Health

## Abstract

**Background:** Inappropriate antibiotic use promotes antibiotic resistance which poses a threat to public health. Outpatient settings are responsible for 80-90% of all antibiotic use, yet up to 50% of these prescriptions may be inappropriate and at least 33% completely unnecessary. To promote outpatient antibiotic stewardship (AS), the Philadelphia Department of Public Health (PDPH) identified outpatient high-volume prescribers (HVPs) in Philadelphia and provided them with peer comparison letters along with evidence-based AS educational resources. **Method:** We identified HVPs using overall and drug specific antibiotic volume and rates from the Centers of Medicare and Medicaid Services (CMS) Part D Prescribers by Provider and by Provider and Drug publicly available datasets for 2021. We restricted analyses to Philadelphia prescribers specializing in internal medicine, family practice, or general practice with antibiotic and beneficiary claim counts ≥ 11. We further restricted the Provider and Drug dataset to prescribers of high-consequence drugs: levofloxacin, ciprofloxacin, and azithromycin. Prescribers with subspecialities where these three drugs are commonly used appropriately were excluded from letter distribution. We analyzed U.S. Census Bureau American Community Survey (ACS) data for overall HVPs on census tract-level to describe health equity demographic characteristics. **Result:** A total of 1,001 prescribers with 67,145 total antibiotic claims, and 306 unique prescribers (77 levofloxacin, 176 ciprofloxacin, and 250 azithromycin) with 37,057 total antibiotic claims met the inclusion criteria in the Provider and the Provider and Drug datasets, respectively. There were 101 overall HVPs, and 89 unique drug-specific HVPs (20 levofloxacin, 45 ciprofloxacin, and 63 azithromycin) based on the highest 10% and 25% of prescribers by antibiotic volume, respectively. The overall HVPs contributed 42.0% of all antibiotic claims. The drug-specific HVPs contributed 60.5% of all antibiotic claims and 55.5% of levofloxacin, ciprofloxacin, and azithromycin claims. These 3 drugs contributed 45.1% of all antibiotic claims. Among the overall and drug-specific HVPs, we sent peer comparison letters to the top 10 by rate per 1,000 beneficiaries (overall) and per 1,000 antibiotic claims (drug-specific), who fell within the following prescribing rates: overall 954-2,714, levofloxacin 84-396, ciprofloxacin 288-723, and azithromycin 496-1,000. **Conclusion:** This initiative identified prescribers at risk for inappropriate use of antibiotics, and empowered these same prescribers to self-reflect on how they prescribe antibiotics. Efforts by local health departments to provide HVPs with peer comparison feedback and AS educational resources may improve the provider knowledge and prescribing habits across different healthcare systems, targeting prescribers at highest risk for misuse or overuse of antibiotics.

**Disclosure:** Jane Gould: Spouse receives salary support- Incyte

